# Diagnostic power of resting‐state fMRI for detection of network connectivity in Alzheimer's disease and mild cognitive impairment: A systematic review

**DOI:** 10.1002/hbm.25369

**Published:** 2021-05-04

**Authors:** Buhari Ibrahim, Subapriya Suppiah, Normala Ibrahim, Mazlyfarina Mohamad, Hasyma Abu Hassan, Nisha Syed Nasser, M Iqbal Saripan

**Affiliations:** ^1^ Department of Radiology, Faculty of Medicine and Health Sciences Universiti Putra Malaysia Serdang Selangor Malaysia; ^2^ Department of Physiology, Faculty of Basic Medical Sciences Bauchi State University Gadau Gadau Nigeria; ^3^ Department of Psychiatry, Faculty of Medicine and Health Sciences Universiti Putra Malaysia Serdang Selangor Malaysia; ^4^ Centre for Diagnostic and Applied Health Sciences, Faculty of Health Sciences Universiti Kebangsaan Malaysia Kuala Lumpur Malaysia; ^5^ Department of Computer and Communication System Engineering Universiti Putra Malaysia Serdang Selangor Malaysia

**Keywords:** accuracy, Alzheimer's disease, classifiers, default mode network, functional MRI, machine learning

## Abstract

Resting‐state fMRI (rs‐fMRI) detects functional connectivity (FC) abnormalities that occur in the brains of patients with Alzheimer's disease (AD) and mild cognitive impairment (MCI). FC of the default mode network (DMN) is commonly impaired in AD and MCI. We conducted a systematic review aimed at determining the diagnostic power of rs‐fMRI to identify FC abnormalities in the DMN of patients with AD or MCI compared with healthy controls (HCs) using machine learning (ML) methods. Multimodal support vector machine (SVM) algorithm was the commonest form of ML method utilized. Multiple kernel approach can be utilized to aid in the classification by incorporating various discriminating features, such as FC graphs based on “nodes” and “edges” together with structural MRI‐based regional cortical thickness and gray matter volume. Other multimodal features include neuropsychiatric testing scores, DTI features, and regional cerebral blood flow. Among AD patients, the posterior cingulate cortex (PCC)/Precuneus was noted to be a highly affected hub of the DMN that demonstrated overall reduced FC. Whereas reduced DMN FC between the PCC and anterior cingulate cortex (ACC) was observed in MCI patients. Evidence indicates that the nodes of the DMN can offer moderate to high diagnostic power to distinguish AD and MCI patients. Nevertheless, various concerns over the homogeneity of data based on patient selection, scanner effects, and the variable usage of classifiers and algorithms pose a challenge for ML‐based image interpretation of rs‐fMRI datasets to become a mainstream option for diagnosing AD and predicting the conversion of HC/MCI to AD.

## Graphical abstract

Resting state fMRI (rs‐fMRI) can aid in the early detection of Alzheimer's disease (AD) and MCI. Deep machine learning (ML) methods can provide a platform for the assessment of the diagnostic accuracy of rs‐fMRI in AD. Much work is yet to be done to achieve homogeneity of the studies before ML can become a mainstream algorithm utilized in clinical practice.

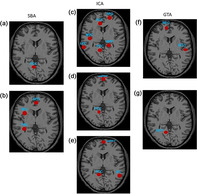

## 1 INTRODUCTION

Alzheimer's disease (AD) is a neurodegenerative disorder that is characterized by a progressive decrease in cognitive function compared to baseline performance level in one or more cognitive domains that can interfere with the ability to independently carry out activities of daily living (American Psychiatric Association, 2013). Resting‐state functional magnetic resonance imaging (rs‐fMRI) is a neuroimaging tool used to study the aberrations in the functional activity of different brain networks, which normally occurs in AD and its prodromal condition, mild cognitive impairment (MCI; X. Chen et al., 2017). The functional connectivity (FC) of brain networks refers to inter‐regional synchrony, as detected from low‐frequency fluctuations in the blood oxygen level dependent (BOLD) fMRI sequence (L. Lee, Harrison, & Mechelli, 2003). FC and other functional features of AD are studied using different molecular imaging techniques such as electroencephalography (EEG), positron emission tomography‐computed tomography (PET/CT), and fMRI. Various radiotracers such as glucose analogs and amyloid detecting radiotracers have been utilized for improving the diagnostic accuracy of detecting AD (Suppiah, Didier, & Vinjamuri, 2019). Of these techniques, fMRI remains the most widely used modality because of the relative simplicity of its usage, inherent safety features, noninvasive nature, and high spatial resolution (Mier & Mier, 2015).

The default mode network (DMN) is the commonest brain network studied by rs‐fMRI and is involved in memory consolidation tasks. It composed of the precuneus (Prec), posterior cingulate cortex (PCC), retro‐splenial cortex, medial parietal cortex (MPC), lateral parietal cortex (LPC), and inferior parietal cortex (IPC), medial prefrontal cortex (mPFC), and the medial temporal gyrus (MTG; Mohan et al., 2016). Fundamentally, AD patients suffer from impaired DMN connectivity (Grieder, Wang, Dierks, Wahlund, & Jann, 2018). There has been consistent evidence of decreased FC in the DMN of AD patients in comparison with HCs, especially between the posterior part of the cerebral cortex (Prec and PCC) and anterior parts, for example, the anterior cingulate cortex (ACC) and mPFC (Brier et al., 2012; Gili et al., 2011; Griffanti et al., 2015). The observed decline in FC in areas within the DMN has also been reported among MCI patients (Cha et al., 2013; Ouchi & Kikuchi, 2012). This indicates that rs‐fMRI detected changes in the DMN can be a noninvasive diagnostic tool for diagnosing AD. In fact, the National Institute on Aging–Alzheimer's Association (NIAAA) has listed rs‐fMRI FC as a potential biomarker of neuronal injury, which is at an early stage of validation (Albert et al., 2011).

There are several methods to analyze rs‐fMRI data, namely, the seed‐based analysis (SBA), the independent component analysis (ICA), and the graph theory analysis (GTA). SBA or small region of interest (ROI) analysis enables temporal correlations to be made between hypotheses‐based predefined seed regions. The SBA investigates the FC of a specific brain region by correlating the brain region's resting‐state time series with the time series of all other regions resulting in the creation of a FC map that identifies the FC of the predefined brain region (T. Jiang, He, Zang, & Weng, 2004). The simplicity and straightforwardness of this seed‐dependent analysis coupled with the clarity of the FC map, makes it popular among researchers (Buckner et al., 2009). Nevertheless, the knowledge from an FC map is restricted to the FC of a pre‐defined region that requires a priori knowledge, making it hard to analyze correlations of FC in whole brain regions.

In contrast to SBA, ICA is free from any predefined seed region selection, which means one does not have to pick a seed or reference area beforehand. Hence, the entire BOLD signal is broken down to produce separate time courses and related spatial maps (De Luca, Smith, De Stefano, Federico, & Matthews, 2005). The resultant components are assumed to be non‐Gaussian signals and are statistically independent of one another. ICA extracts FC information by detecting the patterns of synchronous neural activities between nodes without an a priori knowledge or pre‐existing hypothesis. Thus, the signals from various nodes are temporally filtered from a sample dataset to assess the FC between two independent nodes, which is similar to the “cocktail party effect” (Li, Wang, Chen, Cichocki, & Sejnowski, 2017). The ICA algorithm assumes a set of maximally spatially independent brain components (S), each with associated time course signals (X). The model identifies latent sources whose elements (voxels) have the same time course and thus each component can be considered a measure of the degree to which each voxel is functional connected (correlated) to the component‐time course.

Due to its ability to accommodate whole‐brain FC analysis, ICA is favored over SBA. Nevertheless, the disadvantage of ICA is that there is often difficulty in differentiating useful signals from noise and variations in the separate components. Hence, this causes challenges in making between‐group comparisons using ICA (Fox & Raichle, 2007). Interestingly, both SBA and ICA can ultimately produce similar results if they are run at different experimental set‐ups.

Alternatively, GTA looks at the overall brain network structure with specific spatial information. Here, the BOLD signal undergoes spatial parcellation using a topological mapping of the entire brain, and the relationships between all pairs of activated regions involving “nodes” and “edges” are determined. A “node” is a defined area in the brain, whereas “edge” signifies the direct and indirect links or FC between two defined nodes. Additionally, a “hub” is a node that has an integrative role, which reflects the diversity of a region's cross‐network FCs. A “hub” is defined as a node that has a betweenness centrality or eigenvector centrality (ECi) that is larger than the mean plus two standard deviations (mean + 2 SD) across all nodes in a particular region (Hojjati, Ebrahimzadeh, & Babajani‐Feremi, 2019).

The assessment of the relationship of nodes versus edges of the activated regions is achievable by forming a *p* × *p* square matrix. The fMRI time signal of all participants, “X” is decomposed into a set of maximally independent components, “S” such that both can be transformed to each other via the mixing matrix “A”. Thus, to illustrate the concepts in fMRI, “S” or “components” is a stack of 3D images that will be “mixed” by “A” or “dimensions” that are timepoints by component. Hence, the fMRI signal of all participants, X = A × S, whereby S will be the weighted sum of all the components can be calculated to achieve the series of 3D time point images. In the most common “Dual Regression” approach, first a group ICA is run to estimate the “S” for the whole sample. Then, individual analyses are run to estimate the transformation matrix, “W” for each subject. Notably, the components in “S” then represent the common resting‐state networks, that is, the DMN and visual‐motor networks or physiological noise signals, for example, eyes movement, as well as heart and respiratory motion (J. E. Chen et al., 2020). Finally, one of the networks can be selected to run a multivariate regression on the individual's time courses to estimate group differences for each voxel (Salman et al., 2019).

In this way, the brain is considered as a single complex network where several global and local network topologies such as the path length, modularity of global connectedness, and clustering coefficient can be measured (Rubinov & Sporns, 2010). In GTA, the graph is directly constructed from the Fisher transformed correlation matrix by using each atlas region as the “node” and the *z*‐value as the “edge” weight. The Fisher's *r*‐to‐*z* transformation is applied to the elements of the matrix to improve the normality of the correlation coefficients (Thompson & Fransson, 2016). Whereas in SBA, the extracted time courses of the selected predefined regions are correlated with the time course of all other voxels.

Several studies have analyzed the relationship between the FC of the DMN, Mini‐Mental State Examination (MMSE) test scores, and the development of disease among amnestic MCI (aMCI) and AD patients and compared them with HC subjects (Cha et al., 2013; Liao et al., 2018). Typically, aMCI and AD groups had decreased FC in the left PCC and left parahippocampal gyrus as compared to HC subjects (Liu et al., 2016). Only AD patients were identified with increased FC at the right middle frontal gyrus (MFG), which was interpreted as a compensatory neural mechanism in response to the impairment of the PCC and middle temporal gyrus (MTG; Cha et al., 2013). In the PCC, MTG, and MFG regions, MMSE scores showed significant positive and negative associations with FC (Cha et al., 2013; Liao et al., 2018). Therefore, it is evident that most of the FC disruptions of the DMN occurs in the PCC and MTG (Bai et al., 2009; Zhou et al., 2008). Nevertheless, as the disease progresses, the FC disturbances spread to other brain regions (Damoiseaux, Prater, Miller, & Greicius, 2012). Since the PCC and other DMN hubs are affected in AD and MCI, the DMN may serve as an important biomarker for the classification of patients with AD and MCI. A recent review paper by Badhwar et al. that was published in 2017, studied various patterns of rs‐fMRI detected dysfunctions among patients with AD (Badhwar et al., 2017). Nevertheless, this systematic review did not report on the accuracy of the test to distinguish the disease state.

Subject classifications are made from the FC scores of the rs‐fMRI datasets using machine learning (ML) methods. A commonly applied technique is the support vector machines (SVMs) methods that are applied in patient stratification studies to make inter‐group classifications (Dyrba, Grothe, Kirste, & Teipel, 2015; A. Lee, Ratnarajah, Tuan, Chen, & Qiu, 2015; Park et al., 2017). Another approach is to use Gaussian process logistic regression (GP‐LR) models (Challis et al., 2015). Diagnostic accuracy can be achieved by computing various classifiers that are selected from discriminating features of the multimodal imaging after performing tests using the training dataset (Teipel et al., 2016). A popular type of supervised ML is the support vector machine (SVM) method. SVM has been utilized by various researchers to boost the diagnostic results from multimodal imaging by incorporating multiple kernels in its algorithm (Jin et al., 2020; Q. Zhao, Chen, & Zhou, 2016). Apart from SVM, other more sophisticated algorithms such as convolutional neural networks have been used to discriminate between AD and HC (Qureshi, Ryu, Song, Lee, & Lee, 2019).

The main goal of this review is to examine the benefits and the issues of applying ML algorithms to assess rs‐fMRI datasets for improved diagnostic accuracy of discriminating AD/MCI from HC. We also discuss the limitations of multimodal and multicenter studies, as well as recommend the future direction of research in this field. To the best of the authors' knowledge, this is the only systematic review in the existing literature that is focused on studies that perform rs‐fMRI‐based classification to detect AD and its prodromal stage.

## 2 METHODS AND MATERIALS

We first provide some basic definitions with respect to the classifier methods that are utilized in evaluating the diagnostic accuracy of rs‐fMRI. This is followed by the study protocol of our systematic review. The study protocol includes the study design, search strategy used when screening the articles from the medical databases, selection criteria for identification of eligible articles, assessment of bias, and data extraction. Next, we present the data using tables in Section 3. Afterward, the technicalities of performing ML, for example, SVM, linear regression, logistic regression, and convolutional neural networks, are described in Section 3.3. We also discuss the similarities and differences among the various articles and propose recommendations for future works.

### 2.1 Study design

The systematic review method used to formulate the study design was adopted from Campbell et al. (2015). The results of this review are reported based on the Prepared Reporting Items for Systematic Reviews and Meta‐Analyses (PRISMA) method (Moher, Liberati, Tetzlaff, Altman, & The, 2009).

### 2.2 Search strategy

A preliminary search was conducted to check for existing reviews in the Cochrane central register, Centre for Reviews and Dissemination such as Database of Abstract of Reviews of Effectiveness, National Health Service Economic Evaluation Database and the Health Technology Assessment Database, Turning Research Into Practice (TRIP) Database, and for any on‐going reviews similar to this study. This review protocol has been registered with the International Prospective Register of Systematic Reviews (PROSPERO) with the registration number CRD42020181655.

Scopus, PubMed, DOAJ, and Google Scholar databases were searched for articles using a combination of the keyword using MESH terms. Our search strategy in the various database was as the following‐.

SCOPUS: TITLE‐ABS‐KEY ((“resting‐state functional MRI” OR “resting‐state fMRI” OR rs‐fMRI) AND (Alzheimer's disease OR AD OR mild cognitive impairment OR MCI) AND (accuracy OR classification))

DOAJ TS = ((“resting‐state functional MRI” OR “resting‐state fMRI” OR rs‐fMRI) AND (Alzheimer's disease OR AD OR mild cognitive impairment OR MCI) AND (accuracy OR classification))

PubMed: ((((((“resting‐state functional MRI”) OR “resting‐state fMRI”) OR rs‐fMRI)) AND ((((Alzheimer's disease) OR AD) OR mild cognitive impairment OR MCI)) AND (((((accuracy) OR “classification”))

**FIGURE 1:**
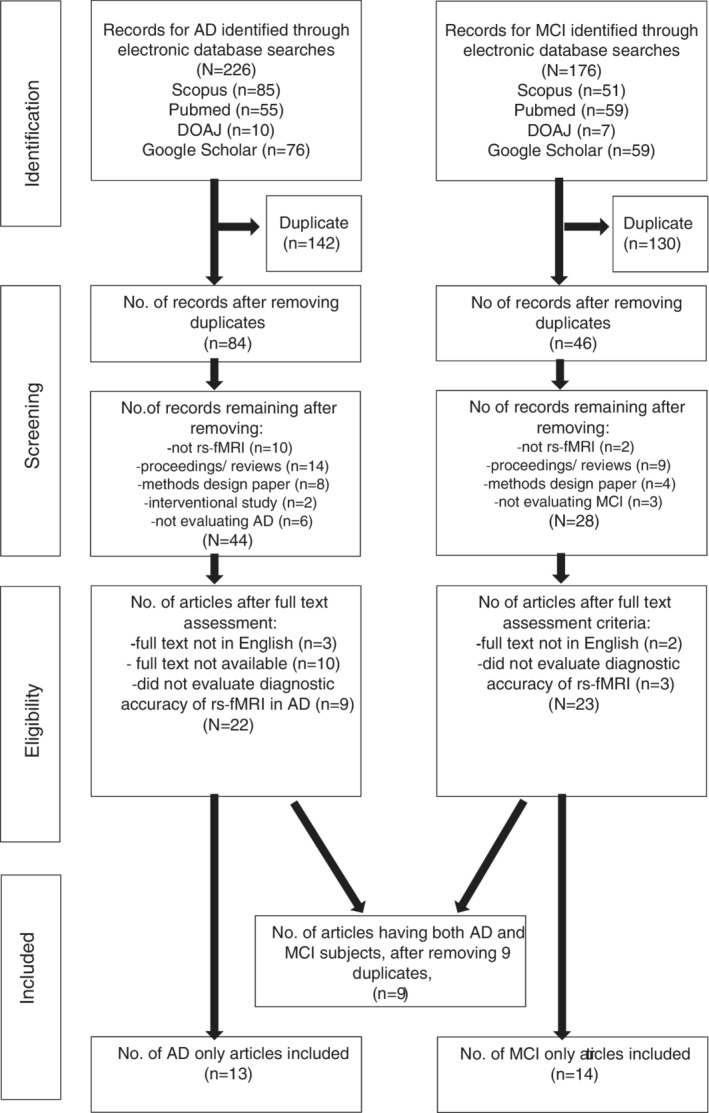
PRISMA flowchart summarizing the literature search and articles selection process

We sourced for relevant published articles through December 3, 2020. The combined articles obtained from the search were screened for duplicates and the resultant articles underwent further screening as highlighted in the subsequent sections (Figure 1).

### 2.3 Criteria for study selection

#### 2.3.1 Inclusion criteria

The review paper included published original articles that met the following criteria: peer‐reviewed articles written in the English language, the articles were sourced from journal publications until December 3, 2020, and the articles included were observational studies of human subjects, which included case–control, cohort, and cross‐sectional studies that utilized rs‐fMRI and the DMN to quantify and correlate FC between AD or MCI with HCs. Furthermore, the articles must have used established AD or MCI diagnostic criteria, for example, Diagnostic and Statistics Manual of Mental Disorders‐IV or V (DSM‐4 or DSM‐5; American Psychiatric Association, 2013) or the revised National Institute of Neurological and Communicative Disorders and Stroke and Alzheimer's Disease and Related Disorders Association (NINCDS‐ADRDA; American Psychiatric Association, 2013).

#### 2.3.2 Exclusion criteria

We excluded articles by the following criteria: (a) Review articles, (b) case reports, (c) case series, (d) articles written in foreign languages, that is, other than the English language, (e) animal studies, and (f) articles with studies using imaging tools other than rs‐fMRI, for example, structural MRI, EEG, MEG, or PET.

### 2.4 Data extraction

We conducted the literature search using the databases mentioned above. Two of the co‐authors (B. I., S. S.) reviewed and independently screened the articles from the search results based on the titles and abstracts for potential inclusion into this review. Only the final screened articles agreed upon by both the authors were considered for the manuscript synthesis. In accordance with the PRISMA protocol, data extracted from each primary study included: author, year, country, number of subjects (patients and controls), age of the subjects, MMSE scores, rs‐fMRI imaging protocol, and analysis method, sensitivity scores, and specificity scores.

### 2.5 Quality assessment

The quality of the methodology of the primary studies used in this review was assessed using the Quality Assessment of Diagnostic Accuracy Study 2 (QUADAS‐2) tool (Beynon et al., 2012). The tool is designed with 14 questions to determine the risk of bias and applicability in terms of patient selection, index test, reference standard, and flow and timing domains. Based on the 13 questions, which served as a reference guide, each domain was rated low, high, or unclear (Table S1). A study was declared to have a low risk of bias in a domain if many of the questions were positively scored for that domain.

## 3 RESULTS

### 3.1 Search results

The summary of the literature search results is in Figure 1. Out of the 226 primary articles obtained for AD studies, 142 duplicates were discarded. Then, 40 nonrelevant articles were removed after screening based on titles and abstracts. Furthermore, 22 articles were removed during the screening of full texts because of noncompliance with our study protocol. Out of the 176 primary articles related to MCI, we removed 130 duplicates and 18 nonrelevant articles during screening. Finally, five articles were removed because they failed to conform to the study inclusion criteria. As a result, 36 articles met our inclusion criteria (13 AD, 14 MCI, and 9 combined AD/MCI articles) and were included in the quantitative synthesis. Thus, the total number of the final included articles was 36 (Balthazar, de Campos, Franco, Damasceno, & Cendes, 2014; Bi, Shu, Sun, & Xu, 2018; Challis et al., 2015; X. Chen et al., 2017; Dai et al., 2012; de Marco et al., 2017; de Vos et al., 2018; Dyrba et al., 2015; Hojjati et al., 2019; Hojjati, Ebrahimzadeh, Khazaee, & Babajani‐Feremi, 2017, 2018; X. Jiang, Zhang, & Zhu, 2014; Jin et al., 2020; Khazaee, Ebrahimzadeh, & Babajani‐Feremi, 2017; Kilian, Bröckel, Overmeyer, Dieterich, & Endrass, 2020; Koch et al., 2012; J. Lee et al., 2015; Y. Li, Wee, Jie, Peng, & Shen, 2014; Lisowska & Rekik, 2019; J. Liu, Pan, Wu, & Wang, 2020; Miao, Wu, Li, Chen, & Yao, 2011; Park et al., 2017; Qian, Zheng, Shang, Zhang, & Zhang, 2018; Qureshi et al., 2019; Schouten et al., 2016; Son, Kim, & Park, 2017; Suk, Wee, Lee, & Shen, 2015; Teipel et al., 2016; Teipel et al., 2017; Wee et al., 2012; Yokoi et al., 2018; Yu et al., 2017; L. Zhang et al., 2020; Y. Zhang, Zhang, Chen, Lee, & Shen, 2017; J. Zhao, Ding, Du, Wang, & Men, 2019; Zheng et al., 2019; Zhu et al., 2014) Overall, the articles had low risk of bias.

### 3.2 Description of the articles included

Tables 1 and 2 summarize the main characteristics of the selected articles, which had assessed the rs‐fMRI diagnostic performance for detecting DMN abnormalities among AD and MCI subjects. The majority (64%) of these articles had ≤20 subjects per group. Five articles (46%) used SBA and three studies (27%) used ICA type of analysis. While one article (9%) utilized both SBA and ICA methods, two articles (18%) used GTA as their method of analysis (Table 3). Interestingly, large percentage of these studies (55%) were conducted in Asia, with China having 5 out of the 7 studies from the region, the other two being from Japan and Korea, respectively.

**TABLE 1:** Sample characteristics of the resting‐state fMRI articles pertaining to Alzheimer's disease studies

Author (year)	Country	Dataset/patient source	Total AD	AD M	AD F	AD age range (mean ± SD) (years)	AD MMSE range (mean ± SD)	Total HC	HC M	HC F	HC age range (mean ± SD) (years)	HC MMSE range (mean ± SD)
Miao et al. (2011)	China	MRI Center of Beijing Normal University	15	6	9	(64 ± 8.27)	0–20 (120 ± 0)	16	7	9	65 ± 9.20	27–30 (29 ± 0)
Dai et al. (2012)	China	Outpatient memory clinic patients at Xuanwu Hospital, Beijing, China	19	NA	NA	(69.56 ± 7.65)	(18.50 ± 3.24)	24	NA	NA	66.55 ± 7.67	(28.59 ± 0.59)
Koch et al. (2012)^a^	Germany	Prospective case control study (site: NA)	15	8	7	58.1–100.2 (76.4 ± 10.3)	NA	21	10	11	56.4–83.0 (68.6 ± 7.3)	NA
Balthazar et al. (2014)	Brazil	Neuropsychology and Dementia Outpatient Clinic (UNICAMP University Hospital)	22	6	16	(73.40 ± 75.67)	(18.86 ± 74.68)	26	6	20	(71.03 ± 76.61)	(28.59 ± 71.86)
Jiang et al. (2014)^a^	USA	ADNI database	34	NA	NA	NA	NA	50	NA	NA	NA	NA
Challis et al. (2015)^a^	UK	Prospective case control study (site: NA)	27	15	12	(68 ± 6.0)	(19 ± 5.0)	39	21	18	(63 ± 9.0)	(26 ± 9.0)
Dyrba et al. (2015)	Germany	German Center for Neuro‐degenerative Diseases (DZNE) Rostock database	28	14	14	(72 ± 7.0)	(24 ± 3.0)	25	12	13	(73 ± 6.0)	(28 ± 1.0)
Lee, Kim, et al. (2015)	South Korea	Prospective case–control study (site: Samsung Medical Center)	61	NA	NA	NA	NA	22	NA	NA	NA	NA
Schouten et al. (2016)^a^	The Netherlands	Subjects scanned at Medical University of Graz	77	31	46	Mild AD: 70.3 ± 7.85 Moderate AD: 66.9 ± 9.06	Mild AD: 24.2 ± 2.07 Moderate AD: 16.6 ± 2.73	173	74	99	66.1 ± 8.71	26.7 ± 5.80
Khazaee et al. (2017)^a^	Iran	ADNI database	34	16	18	(72.54 ± 7.02)	(21.24 ± 3.37)	45	19	26	(75.90 ± 6.79)	(28.95 ± 1.56)
Park et al. (2017)	South Korea	Asan Medical Centre database ADNI database	41 16	13 9	28 7	(71.2 ± 7.5) (73.6 ± 4.1)	(17.2 ± 5.4) (19.4 ± 5.5)	22 19	9 11	4 8	60–80 (67.9 ± 4.5) (72.5 ± 7.9)	(29.3 ± 1.6) (29.5 ± 0.8)
Son et al. (2017)^a^	South Korea	ADNI database	30	12	18	(74.00 ± 7.46)	(19.40 ± 3.62)	35	12	23	(76.06 ± 7.38)	(29.43 ± 1.14)
Teipel et al. (2016)	Germany	Datasets from four different centers of the “German resting‐state initiative for diagnostic biomarkers” (psymri.org)	53	22	31	(72.4 ± 8.8)	(22.5 ± 4.4)	118	57	61	(70.4 ± 6.2)	(28.8 ± 1.0)
Teipel et al. (2017)^a^	Germany	Datasets from five different centers of the “German resting‐state initiative for diagnostic biomarkers” (psymri.org)	84	38	46	(72.0 ± 9.0)	(22.4 ± 4.4)	151	69	82	(69.0 ± 7.8)	(28.9 ± 1.0)
Bi et al. (2018)	China	ADNI database	25	12	13	(74.59 ± 7.03)	NA	35	15	20	(77.09 ± 6.69)	NA
de Vos et al. (2018)	The Netherlands	Medical University of Graz as a part of the prospective registry on dementia (PRODEM)	77	31	46	47–83 (68.6 ± 8.6)	10–28 (20.4 ± 4.5)	173	74	99	47–83 (66.1 ± 8.7)	22–30 (27.5 ± 1.8)
Yokoi et al. (2018)	Japan	Patient subjects from outpatient clinic of the Department of Neurology, Nagoya University Hospital, and Dementia Center of Meitetsu Hospital in Nagoya.	23	4	19	(68.6 ± 7.8)	(23.6 ± 2.8)	24	8	16	(65.4 ± 7.3)	(29.4 ± 1.0)
Hojjati et al. (2019)^a^	Iran	ADNI database	34	16	18	72.54 ± 7.02	21. 24 ± 3.37	49	21	28	74.47 ± 7.68	29.35 ± 1.63
Qureshi et al. (2019)	South Korea	Part of a large cohort enrolled at National Dementia Research Center, Chosun University, Gwangju, South Korea	Very mild to mild AD: 77 Moderate to severe AD: 49	Very mild to mild AD: 47 Moderate to severe AD: 17	Very mild to mild AD:30 Moderate to severe AD:32	Very mild to mild AD: (73.57 ± 6.49) Moderate to severe AD: (73.61 ± 4.76)	Very mild to mild AD: (23.84 ± 3.90) Moderate to severe AD: (15.49 ± 4.87)	–	–	–	–	–
Zhao et al. (2019)	China	ADNI database	45	22	23	(72.6 ± 7.1)	(21.24 ± 3.44)	45	20	25	(74.3 ± 8.4)	(28.45 ± 1.82)
Zheng et al. (2019)	China	Subjects scanned at Xuanwu Hospital, China	40	18	22	(65 ± 10.0)	8–20 (14.00 ± 6.00)	30	15	15	(64 ± 8.0)	26–30 (28.00 ± 2.00)
Jin et al. (2020)^a^	China	Multicenter rs‐fMRI study (6 different scanners) F18‐AV‐45 PET scans from ADNI database were used for correlation analysis	252 291	NA	NA	NA	NA	215 334	NA	NA	NA	NA

Abbreviations: AD, Alzheimer's disease; ADNI, Alzheimer's Disease Neuroimaging Initiative; *BIAC, Duke‐UNC Brain Imaging and Analysis Center (BIAC), Durham, North Carolina, USA; HC, healthy control; NA, not available.

^a^ Studies that have datasets of both AD and MCI subjects.

**TABLE 2:** Sample characteristics of the resting‐state fMRI articles pertaining to MCI studies

Author (year)	Country	Dataset/patient source	Total MCI	MCI M	MCI F	MCI age range (mean ± SD)	MCI MMSE range (mean ± SD)	Total HC	HC M	HC F	HC age range (mean ± SD)	MMSE range (mean ± SD)
Koch et al. (2012)^a^	Germany	Prospective case control study (site:NA)	38	7	10	60.4–89.0 (74.6 ± 7.0)	NA	21	10	11	56.4–83.0 (68.6 ± 7.3)	NA
Wee et al. (2012)	USA	*BIAC	10	5	5	(74.2 ± 8.6)	(28.4 ± 1.5)	17	8	9	72.1 ± 8.2	(29.4 ± 0.9)
Jiang et al. (2014)^a^	USA	ADNI database	100	NA	NA	NA	NA	50	NA	NA	NA	NA
Y. Li et al. (2014)	USA	*BIAC	12	NA	NA	NA	NA	25	NA	NA	NA	NA
Zhu et al. (2014)	USA	*BIAC	Dataset 1:10 Dataset 2:12	− 5 − 5	− 5 − 7	55–84 (74.2 ± 6 8.6) 68–84 (78.1 ± 4.8)	26–30 (28.4 ± 1.5) 19–28 (25.5 ± 2.5)	Dataset 1:10 Dataset 2:12	− 1 − 2	− 9 − 10	55–82 (67.7 ± 8.1) 66–81 (72.3 ± 5.1)	29–30 (29.8 ± 0.4) 25–30 (28.3 ± 1.7)
Challis et al. (2015)^a^	UK	Prospective case control study (site: NA)	50	5	22	(66 ± 7.0)	(26 ± 4.0)	39	21	18	(63 ± 9.0)	(26 ± 9.0)
Lee, Ratnarajah, et al. (2015)^a^	South Korea	Prospective case control study (site: Samsung Medical Center)	37	NA	NA	NA	NA	22	NA	NA	NA	NA
Suk et al. (2015)	USA	*BIAC	12	6	6	75.0 ± 8.0	28.5 ± 1.5	25	9	16	72.9 ± 7.9	29.3 ± 1.1
Chen et al. (2017)	USA, South Korea	ADNI database	54	NA	NA	NA	NA	54	NA	NA	NA	NA
de Marco et al. (2017)	Italy	Subject from a Venetian lagoon	50	25	25	(73.86 ± 6.31)	(27.46 ± 1.92)	50	19	31	(69.54 ± 5.88)	(28.98 ± 1.32)
Hojjati et al. (2017)	Iran	ADNI database	MCI‐C:18 MCI‐NC:62	MCI‐C: 11 MCI‐NC:28	MCI‐C:7 MCI‐NC:34	MCI‐C: 73.6 ± 15.7 MCI‐NC: 73.0 ± 16.3	MCI‐C: 26.0 ± 2.0 MCI‐NC: 27.0 ± 3.0	–	–	–	–	–
Khazaee et al. (2017)^a^	Iran	ADNI database	89	43	46	(71.77 ± 7.78)	(27.56 ± 2.20)	45	19	26	(75.90 ± 6.79)	(28.95 ± 1.56)
Son et al. (2017)^a^	South Korea	ADNI database	40	19	21	(74.30 ± 7.67)	(27.55 ± 2.15)	35	12	23	(76.06 ± 7.38)	(29.43 ± 1.14)
Teipel et al. (2017)^a^	Germany	Datasets from five different centers of the “German resting‐state initiative for diagnostic biomarkers” (psymri.org)	115	56	59	(72.6 ± 8.0)	(26.7 ± 1.8)	151	69	82	(69.0 ± 7.8)	(28.9 ± 1.0)
Yu et al. (2017)	China, USA	ADNI database	50	NA	NA	NA	NA	49	NA	NA	NA	NA
Zhang et al. (2017)	USA	ADNI database	29	16	13	(73.7 ± 4.8)	NA	30	13	17	(74.4 ± 5.7)	NA
Hojjati et al. (2018)	Iran	ADNI database	MCI‐C:18 MCI‐NC:62	MCI‐C: 11 MCI‐NC: 28	MCI‐C: 7 MCI‐NC: 34	MCI‐C: (73.6 ± 15.7) MCI‐NC: (73.0 ± 16.3)	MCI‐C: (26.0 ± 2.0) MCI‐NC: (27 ± 3.0)	–	–	–	–	–
Qian et al. (2018)	China	ADNI databases	37	16	21	72.35 ± 8.78	27.70 ± 1.97	32	14	18	75.63 ± 5.70	28.65 ± 2.01
Hojjati et al. (2019)^a^	Iran	ADNI database	MCI‐C:25 MCI‐NC:69	MCI‐C:14 MCI‐NC:32	MCI‐C:11 MCI‐NC:37	MCI‐C: 73.02 ± 11.80 MCI‐NC: 72.95 ± 11.92	MCI‐C: 26.64 ± 1.85 MCI‐NC: 27.57 ± 2.21	49	21	28	74.47 ± 7.68	29.35 ± 1.63
Lisowska and Rekik (2019)	UK	ADNI database	Early MCI: 42	NA	NA	70.4 ± 7.5	NA	HC: 42	NA	NA	74.1 ± 6.7	NA
Jin et al. (2020)^a^	China	Multicenter rs‐fMRI study (6 different scanners) F18‐AV‐45 PET scans of AD and HC from ADNI database were used for correlation analysis	221	NA	NA	NA	NA	215	NA	NA	NA	NA
Liu et al. (2020)	China	ADNI database	Late MCI:105 Early MCI:105	Late MCI: 35 Early MCI: 49	Late MCI: 70 Early MCI: 56	Late MCI: 75.8 ± 6.3 Early MCI: 76.3 ± 5.4	Late MCI: 26.6 ± 2.2 Early MCI: 27.5 ± 1.8	105	54	51	77.1 ± 6.3	29.1 ± 1.1
Zhang et al. (2020)	UK	ADNI database	82	36	57	71.61 ± 5.1	28.88 ± 1.46	93	46	36	70.47 ± 5.91	27.89 ± 1.82

Abbreviations: ADNI, Alzheimer's disease Neuroimaging Initiative; *BIAC, Duke‐UNC Brain Imaging and Analysis Center (BIAC), Durham, North Carolina, USA; MCI, mild cognitive impairment; MCI‐C, MCI converter; MCI‐NC, MCI non‐converter; NA, not available; F18‐AV‐45, amyloid PET tracer.

^a^ Studies that have datasets of both AD and MCI subjects.

**TABLE 3:** Diagnostic performance of classification using various machine learning methods to discriminate between AD and healthy control subjects

Author (year)	*N* AD		*N* HC	Method of analysis	Diagnostic accuracy measurement	Significant findings/ROIs	Sensitivity (%)	Specificity (%)	Accuracy (%)
Miao et al. (2011)	15		16	ICA with 59 components for the AD group determined for PCA	Granger causality modeling of DMN hubs ROC curve (cutoff 0.647)	PCC IPC mPFC LTC HIPP LTC	80.00	81.25	NA
Dai et al. (2012)^a^	22		16	Structural MRI, which was used to measure regional gray matter volume rs‐fMRI, using amplitude of low‐frequency fluctuations (ALFF), regional homogeneity (ReHo), and regional functional connectivity strength (RFCS)	Multi‐classifier (M3) based on four maximum uncertainty linear discriminant analysis base classifiers	90 ROIs Discriminative features for classification: DMN (mPFC, PCC, HIPP, and paraHIPP), occipital regions (fusiform gyrus, inferior, and middle occipital gyrus), and subcortical (amygdala and pallidum of lenticular nucleus)	87.50	90.91	89.47
Koch et al. (2012)	15		21	Rs‐fMRI SBA ICA	Discriminant analyses group classifications: Time course correlation analyses (TCC) ICA determination of magnitude of coactivation between nodes Combination of both approaches	DMN and non‐DMN nodes	TCC: 86.7 ICA: 53.3 Combined: 100	TCC: 95.2 ICA: 71.4 Combined: 95.2	TCC: 91.7 ICA: 63.9 Combined: 97.2
Balthazar et al. (2014)	22		26	Rs‐fMRI SBA of DMN and WCP	ROC curve analysis	PCC	DMN, cutoff *z*‐score of 0.267:77.3 WCP, cutoff *z*‐score of 0.195:72.7	DMN, cutoff *z*‐score of 0.267:70.0 WCP, cutoff *z*‐score of 0.195:70.0	NA
Jiang et al. (2014)^a^	35		50	Rs‐fMRI using RSNs derived from ICA. Sparse representation of fMRI signals and identification of 10 RSNs	Six types of features (SOR, FC‐RSNs, FC‐D, ET‐FC, ET‐CDRSNs, and CDC) in the RSNs	RSNs#1, #2, and #3: “visual” cortex, which includes medial, occipital pole, and lateral visual areas, RSN #4: DMN, RSN #5: cerebellum, RSN #6: “sensorimotor” network, RSN #7: “auditory” system, RSN #8: ECN, which includes the ACC and the paracingulate regions, RSNs #9 and #10 show networks that have strong lateralization, which includes the middle frontal, orbital, and superior parietal areas	CFS: 94.12	CFS: 94.12	CFS: 94.12
Challis et al. (2015)^a^	27		39	rs‐fMRI dataset post‐processed using SBA to include 82 anatomically distinct ROIs based on a priori selection	Gaussian process logistic regression (GP‐LR) model	This dataset also included MCI patients and the classification was aimed at discriminating between AD and MCI	AD versus MCI: 88.0	AD versus MCI: 93.0	AD versus MCI: 91.0
Dyrba et al. (2015)	28		25	Fiber tract integrity as measured by DTI GMV derived from structural MRI rs‐fMRI dataset derived analyzed using GTA measures of “local clustering coefficient” and “shortest path length”	The parameters were used as classifiers and ROC curve analyses were conducted for single modality parametric assessment and multimodal SVM assessment combinations using multiple kernel SVM method. SVM algorithm was validated using the LOOCV method		rs‐fMRI: 82.0 DTI: 86.0 GMV: 82.0 DTI and GMV: 79.0 3 combined: 82.0	rs‐fMRI: 64.0 DTI: 84.0 GMV: 80.0 DTI and GMV: 92.0 3 combined: 76.0	rs‐fMRI: 74.0 DTI: 85.0 GMV: 81.0 DTI and GMV: 85.0 3 combined: 79.0
Lee, Kim, et al. (2015)^a^	61		22	59 brain neural pathways based on a priori knowledge were analyzed 116 nodes were identified and the FC between nodes at paired brain regions was measured by the strength of the linear relationship depicted by *r*	Three linear classifiers: Naïve Bayesian (NB); logistic regression; and SVM One decision trees classifier: RF Diagnostic performances were evaluated on a pathway‐based approach and a region‐based approach	SVM classification model gave the best diagnostic accuracies for discriminating AD from HC, for both the pathway‐based approach and a region‐based approach.	SVM in Pathway‐based approach: 85.0 Region‐based approach: 78.0	SVM in Pathway‐based approach: 73.0 Region‐based approach: 69.0	SVM in Pathway‐based approach: 79.0 Region‐based approach: 74.0
Schouten et al. (2016)	77		173	Structural MRI analysis: GMD, WMD were calculated DTI analysis: FA, MD values Temporal concatenation ICA	Six feature vectors from the three modalities with a logistic elastic net regression for classification	Optimal combination of multimodal procedure consisted of GMD, WMD, FA, MD, and sparse partial correlations between functional rs‐fMRI networks (PC).	Multimodal procedure results: Mild AD: 72.1 Moderate AD: 81.3	Multimodal procedure results: Mild AD: 93.5 Moderate AD: 95.6	Multimodal procedure results: Mild AD: 89.6 Moderate AD: 93.0
Khazaee et al. (2017)^a^				Graph measure of rs‐fMRI dataset Time series of voxels within each of 264 ROIs were averaged to generate a representative signal for each ROI Binary directed connectivity matrix for each subject was used to calculate 13 graph measures	Multivariate Granger causality is performed by including more than two variables in a MVAR model Types of classifiers used: LDA, KNN, decision trees, SVM, and naïve Bayes classifier were used to discriminate between the features of MCI and HC	Naïve Bayes classifier achieved the best performance to discriminate between the features of MCI and HC, with the top features that had the most discriminating ability rising from nodes of the DMN	SVM: 51.55 Naïve Bayes: 81.8	SVM: 97.7 Naïve Bayes: 100	SVM: 71.95 Naïve Bayes: 93.29
Park et al. (2017)	57		41	Cortical thickness of the mPFC, STG, SMG, and so on were evaluated FC of the nodes were evaluated using ICA method	Diagnostic accuracy of the combination of mPFC‐PCC FC with the regional CThk abnormalities versus the CThk of the bilateral medial temporal lobes were calculated, using these classifiers, and applying SVM	AD had a significantly lower *r* value for mPFC‐PCC FC than HCs Adding the CThk of the STG and SMG of the left cerebral hemisphere to mPFC‐PCC FC yielded a greater diagnostic accuracy (combined SVM1)	Combined SVM1: 68.7	Combined SVM1: 94.7	Combined SVM1: 91.7
Son et al. (2017)^a^	30		35	10 subcortical regions (thalamus L/R, putamen L/R, hippocampus L/R, caudate L/R, and amygdala L/R) to identify any presence of regional volume atrophy The rs‐fMRI dataset was analyzed using graph theory by using nodes from predefined ROIs and unweighted edges in a square matrix ECi was used as a connectivity measure of the functional networks	Random forest (RF) classifier using identified regional volume and ECi values of network functional connectivity as features.	The classifier chose among three possible outcomes and gave improved accuracy. Functional degeneration increased as the disease progressed from HC to MCI to AD, evidenced by seven regions (HIPP L/R, thalamus L/R, putamen L/R, and amygdala L) that showed significant differences in volume between HC and AD. Putamen L showed significant differences in ECi between MCI and AD, whereas HIPP L showed significant ECi differences between HC and AD.	NA	NA	RF classifier accuracy in distinguishing among HC, MCI, and AD Using cortical volume and ECi of identified regions: 53.33
Teipel et al. (2016)	53		118	For each individual, the time series of signal was extracted for each of the 84 functionally defined ROIs of the Greicius atlas. Pearson's correlation coefficients (*r*) were computed for the 3,486 possible pairs of correlations between these 84 ROIs. Using Fisher's Z transform, the *r*'s of the signal time courses were adjusted to be normally distributed.	Two regression models were utilized: (i) bidirectional stepwise unpenalized LR using the function step in R (The R Foundation for Statistical Computing); (ii) penalized LR models with an elastic net penalty. The selected features from elastic net were mainly from the dorsal part of the DMN functional network. The accuracy of prediction was determined by AUC of the ROC curves.	More accurate group discrimination between AD cases and HCs and more homogeneous feature selection from rs‐fMRI data when using regularized LR with an elastic net penalty compared with a classical stepwise LR. Decreased functional connectivity in AD in the STG, a region that is involved in language processing, and prefrontal parts of the salience network, prefrontal and parietal components of executive control networks, as well as the medial occipital gyrus as part of the ventral visual stream involved in object recognition and recognition of limb movements	NA	NA	Multi‐center study, cross‐validated accuracy from elastic net regression: 80.0
Teipel et al. (2017)^a^	84		151	Individual gray matter volumes of the HIPP were extracted ROIs of brain regions that showed significant group differences in the voxel‐based comparisons of AD and HC subjects were defined	A block‐wise cross validation with repeated random sampling, based on Gaussian‐distributed random numbers generated in R was used to estimate the accuracy of group discrimination for each modality and analysis technique. The dataset was split by a ratio of 3:2 for the training data and the test data, respectively. LR analysis was applied and classification accuracy and AUC were recorded.	FC of the PCC was smaller in AD compared to HC AD versus HC demonstrated peak areas of group effects at the mid temporal cortex, ACC, and inferior parietal cortex The effect of scanner on FC, in this multi‐center study was determined, using the diagnosis as fixed effect covariate and scanner as random effect covariate. Framewise displacement (head motion) showed comparable displacements across sites, for example, cognitively impaired patients showed slightly more head motion than controls. The foreground‐to‐background energy ratio, the fractional ALFF in PCC, and the mean FC between PCC and anterior mPFC indicated no outlying center. tSNR was significantly different between certain centers.	NA	NA	Pooled accuracy: 76.1
Bi et al. (2018)	25		35	45 ROIs in each hemisphere were utilized from the rs‐fMRI dataset, the time series of each region was obtained and the *r* of every two regions was calculated. GTA of 170 weighted functional connections were analyzed	Random SVM clusters are used for classification and feature selection.	Abnormal FC of AD compared with HC are mainly concentrated in frontal lobe and cingulate cortex	NA	NA	94.44
de Vos et al. (2018)	77		173	Features that were extracted from the rs‐fMRI dataset: Static and dynamic FC were extracted ALFF was calculated for each subject GTA metrics were utilized to analyze the FC matrices Whole brain FC with the HIPP as a hub was calculated using regression analysis ECi was computed	Elastic net regression was utilized for classification. AUC of ROC curves were evaluated to determine the accuracy of discriminating AD from HC	FC with the default mode network (AUC =0.70) and the executive control network (AUC = 0.71). FC with the left (AUC = 0.59) and right (AUC = 0.51) hippocampus result in poor classification performances and ECi mapping results in moderate classification performance (AUC = 0.69). FC dynamics/dynamic state FC with SD of 70 × 70 sparse partial correlation FC matrix provided the best accuracy for discriminating between AD and HC	FC dynamics: 83.0	FC dynamics: 73.0	FC dynamics: 78.0
Yokoi et al. (2018)	23		24	After injecting 185 MBq of THK‐5351 or 555 MBq of PiB, THK5351 or PiB PET images were acquired for all subjects Standardized uptake values (SUV) images were acquired by normalizing tissue radioactivity Concentration of PiB by injected dose and body weights, with the cerebellum as a reference point to give the SUV ratio (SUVR). If the regions of the neocortices had SUVR >1.5, then the subjects were considered as “Aβ positive” ICA analysis was used to obtain group RSNs Two subjects were subjected to post‐mortem and autopsy samples of the brain were evaluated for phosphorylated tau aggregations, senile plaques, and Aβ deposition.	Seed‐based analysis of the precuneus/PCC and dorsolateral prefrontal cortex (DLPFC) was performed. Dual regression analysis was utilized to compute subject‐specific RSNs. Statistical analysis of the different RSNs was performed using nonparametric permutation testing to identify significant differences in FC between the AD group and HC.	The most significant difference in THK5351 retention between early AD and healthy controls was observed in the bilateral precuneus/PCC and the left DLPFC. In early AD, the intrinsic connectivity of precuneus/PCC significantly decreased In the left middle occipital gyrus, left STG, left amygdala/HIPP, and right fusiform gyrus	82.6	79.1	NA
Hojjati et al. (2019)^a^	34		49	The adjacency matrix was calculated using the Pearson's correlation between the time series of the fMRI signals of all pairs of 160 ROIs of Dosenbach atlas Converted the weighted adjacency matrices to binary ones by applying an optimal threshold	Discriminant correlation analysis (DCA) and sequential feature collection (SFC) were utilized. The SFC algorithm sorts all features using the multivariate MRMR feature selection algorithm. The MRMR feature selection algorithm selects features that have maximal statistical dependency based on mutual information by considering relevant and redundant features simultaneously. The selected features were used to train and cross‐validate an SVM to classify four groups of subjects (AD, MCI‐C, MCI‐NC, and HC) in the train/cross‐validation set.	SFC outperforms DCA for feature selection in three‐ and four‐group classification with an extra accuracy >7%	Four group classification (AD, MCI‐C, MCI‐NC, HC): 46.1 Three group classification (AD, MCI‐C, MCI‐NC): 52.3	Four group classification (AD, MCI‐C, MCI‐NC, HC): 85.0 Three group classification (AD, MCI‐C, MCI‐NC): 91.1	Four group classification (AD, MCI‐C, MCI‐NC, HC): 65.0 Three group classification (AD, MCI‐C, MCI‐NC): 72.0
Qureshi et al. (2019)	Very mild to mild AD: 77 Moderate to severe AD: 49		‐	rs‐fMRI dataset was used to extract FC features using ICA	Automated severity classification with three‐dimensional convolutional neural networks (3D‐CNNs) based on deep learning	CDR‐based novel classification of rs‐fMRI can be accepted as an objective severity index. The medial frontal, sensory‐motor, executive control, left dorsal attention, lateral visual‐related, cerebellar, medial visual‐related, auditory related, frontoparietal, and right dorsal attention networks have high ranks and statistical differences between the two groups	89.6	94.6	92.3
Zhao et al. (2019)	45		45	Rs‐fMRI dataset static FC and dynamic FC, were tested using different p‐value and corresponding accuracy, by selecting the feature subset with the highest accuracy	SVM classification model was utilized	The performance of feature subsets selected from sWGFC was better than sGFC, and the performance of feature subsets selected from dWGFC was better than dGFC	dWGFC: 84.44	dWGFC: 77.78	dWGFC: 81.11
Zheng et al. (2019)	40		30	ALFF and FC utilizing SBA were performed Regional cerebral blood flow (rCBF) was assessed using arterial spin labeling (ASL) sequence	Interregional correlation analysis was performed with regards to regional FC and rCBF, rCBF and ALFF analysis of the Precuneus/PCC as a biomarker was also conducted	The combined rCBF and ALFF values of Precuneus/PCC as a biomarker to differentiate the two groups reached good diagnostic accuracy to discriminate AD from HC	85.3	88.5	NA
Jin et al. (2020)^a^	252		215	Four measures of functional brain activity and connectivity derived from each individual's rs‐fMRI data were used: amplitude of local brain activity (AM), regional homogeneity (ReHo), functional connectivity strength (FCS), and whole‐brain connectivity	Linear SVM classifier to predict individual diagnostic status, for all patients from the six MRI centers, was utilized, combining classifiers from MMSE scores, AM, ReHo, FCS, and whole‐brain connectivity	AD was associated with significantly reduced FC and local activity in the DMN, basal ganglia, and cingulate gyrus, along with increased FC or local activity in the prefrontal lobe and HIPP	Pooled results based on training dataset: 82.0	Pooled results based on training dataset: 60.0	Pooled results based on training dataset: 70.0

Abbreviations: ACC, anterior cingulate cortex; AD, Alzheimer's disease; AUC, area under the curve; CDC, common dictionary distribution; CFS, correlation‐based feature selection; CThk, cortical thickness; dGFC, dynamic functional connectivity within gray matter; DTI, diffusion tensor imaging; dWGFC, dynamic functional connectivity between WM and GM; ECi, eigenvector centrality; ECN, executive control network; ET‐CDRSNs, entropy of component distribution within RSNs; ET‐FC, entropy of functional connectivity; FA, fractional anisotropy; FC, functional connectivity; FC‐D, functional connectivity within dictionary; FC‐RSNs, functional connectivity within RSNs; GM, gray matter; GMD, gray matter density; GMV, gray matter volume; GTA, graph theory analysis; GTA, graph‐theoretical analysis; HC, healthy control; HIPP, hippocampus; LOOCV, leave‐one‐out cross‐validation; LR, logistic regression; mPFC, medial prefrontal cortex; MRMR, minimal redundancy maximal relevance; PCA, principal component analysis; PCC, posterior cingulate cortex; *r*, Pearson's correlation coefficient; RF, random forest; ROC, receiver operating characteristic; ROC, receiver operating characteristics; ROIs, regions of interest; RSN, resting‐state networks; SBA, seed‐based analysis; sGFC, static functional connectivity within gray matter; SMG, supramarginal gyrus; SOR, spatial overlapping rate; STG, superior temporal gyrus; sWGFC, static functional connectivity between WM and GM; tSNR, temporal signal‐to‐noise ratio; WCP, whole cortical positive *z*‐average; WM, white matter; WMD, white matter density.

^a^ Studies that have datasets of both AD and MCI subjects.

### 3.3 Types of machine learning methods utilized to classify Alzheimer's disease subjects

Machine learning (ML) is a form of artificial intelligence application that utilizes computer algorithms. The basis of ML is dependent upon the ability of the computer program to leverage algorithms. Hence, ML can automatically learn and improve from experience gathered on an independent training dataset based on statistical models. There are various computer programming languages for ML such as Python, Java, R, and JavaScript. These programs can perform ML in any one of the three types of ML, which are supervised learning, unsupervised learning, and reinforcement learning. Supervised learning is the basic type of ML that is frequently used in classification for diagnostics and automated image interpretation. This type of learning can also be used to predict an outcome, such as the occurrence of a disease by using regression analysis. To this end, the training dataset needs to be labeled correctly and provides the algorithm with a fundamental concept of the problem, solution, and data points to be dealt with.

**FIGURE 2:**
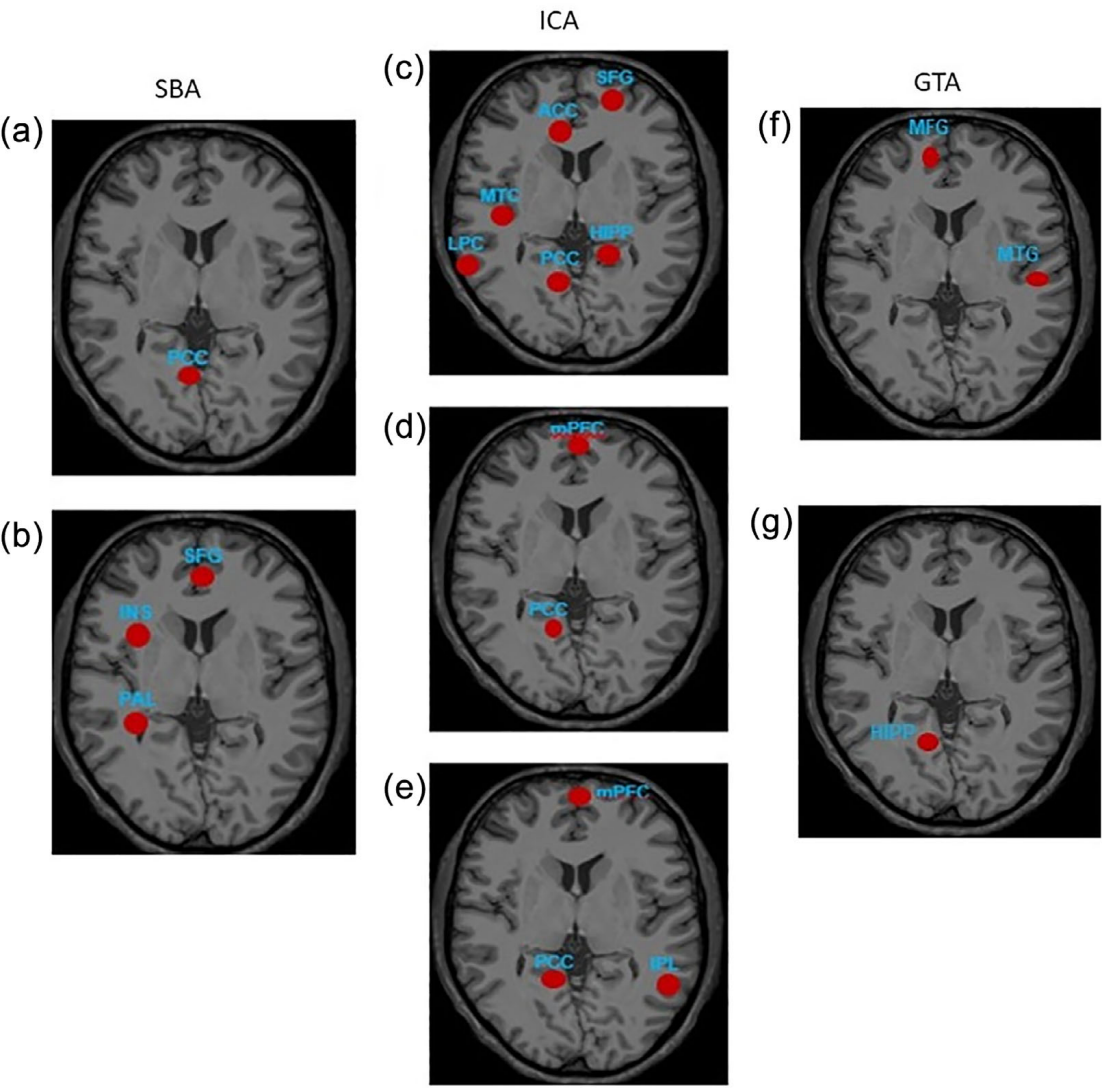
Regional functional connectivity on BOLD fMRI in AD and MCI brain [ROIs sourced from (a) Balthazar et al. (2014) showing AD brain, (b) Dai et al. (2012) showing AD brain, (c) Koch et al. (2012) showing MCI brain, (d) Li et al. (2011) showing MCI brain, (e) Park et al. (2017) showing AD brain, (f) Qian et al. (2018) showing MCI brain, and (g) de Vos et al. (2018) showing AD brain]. Abbreviations: *ACC, anterior cingulate cortex; DLPFC, dorsolateral prefrontal cortex; GTA, graph theory analysis; HIPP, hippocampus; ICA, independent component analysis; INS, insular; IPC, inferior parietal cortex; LPC, lateral parietal cortex; MFG, medial frontal gyrus; MOG, medial orbitofrontal gyrus; mPFC, medial prefrontal cortex; MTC, medial temporal cortex; MTG, medial temporal gyrus; PCC, posterior cingulate cortex; Prec, precuneus; SBA, seed‐based analysis; SFG, superior frontal gyrus

In rs‐fMRI, the data points are the “nodes” and the FC between the “nodes” are called “edges”. The probability of the number of connections or “edges” arising from the nodes gives the weightage of the FC. A Bayesian network (BN) is a probabilistic graphical model that represents a joint probability distribution over a set of variables. Nodes in a BN graph represent variables of interest, and edges represent the probabilistic associations among variables. In a BN, each node has a conditional‐probability distribution, which quantifies the association between that variable and the variables with which it is associated. There are different types of BNs, based on constraints on allowed model structures. Several articles included in our review demonstrated the diagnostic power of rs‐fMRI in detecting DMN abnormalities for distinguishing AD and MCI patients from HCs, based on the brain regions that demonstrated weaker FC (Tables 3 and 4; Balthazar et al., 2014; Dai et al., 2012; de Vos et al., 2018; Koch et al., 2012; Y. Li et al., 2014; Miao et al., 2011; Park et al., 2017; Yokoi et al., 2018; Zheng et al., 2019). Examples of salient regions of the DMN that demonstrated impaired FC among AD subjects in selected studies are shown in Figure 2.

**TABLE 4:** Diagnostic performance of classification using various machine learning methods to discriminate between MCI and healthy control subjects

Author (year)	*N* MCI	*N* HC	Imaging parameters/method of analysis	Diagnostic accuracy measurement	Significant findings/ROIs	Sensitivity (%)	Specificity (%)	Accuracy (%)
Koch et al. (2012)^a^	17	21	Rs‐fMRI SBA ICA	Discriminant analyses group classifications: Time‐course correlation analyses (TCC) ICA determination of magnitude of coactivation between nodes Combination of both approaches	DMN and non‐DMN nodes	Combined TCC and ICA: 64.7	Combined TCC and ICA: 95.2	Combined TCC and ICA: 81.6
Wee et al. (2012)	10	17	Rs‐fMRI DTI integration (parcellated into 45 regions per hemisphere) using AAL ROIs	Multimodal data fusion using multiple‐kernel SVM Evaluated classification accuracy and the AUC of ROC curve	Integration of biomarkers from structural MRI, rs‐fMRI and DTI enable multimodal analysis of network connectivity Salient areas for accurate classification include the PFC, orbitofrontal cortex, ACC, and PCC	rs‐fMRI:70.0 Proposed combined rs‐fMRI and DTI:100.0	rs‐fMRI:70.59 Proposed combined rs‐fMRI and DTI:94.12	rs‐fMRI:70.37 Proposed combined rs‐fMRI and DTI:96.30
Jiang et al. (2014)^a^	100	50	Rs‐fMRI using RSNs derived from ICA. Sparse representation of fMRI signals and identification of 10 RSNs	Six types of features (SOR, FC‐RSNs, FC‐D, ET‐FC, ET‐CDRSNs, and CDC) in the RSNs	RSNs#1, #2, and #3: “visual” cortex, which includes medial, occipital pole, and lateral visual areas, RSN #4: DMN, RSN #5: cerebellum, RSN #6: “sensorimotor” network, RSN #7: “auditory” system, RSN #8: ECN, which includes the ACC and the paracingulate regions, RSNs #9 and #10 show networks that have strong lateralization, which includes the middle frontal, orbital and superior parietal areas	CFS: 94.00	CFS: 90.00	CFS: 92.00
Li et al. (2014)	12	25	Rs‐fMRI using ICA	Sparse effective connectivity using Granger causality MAR modeling using OLS algorithm SVM with nonlinear kernel	MCC and PCC regions are causally influenced by the IFG ACC regions LING and CAU regions are only influenced by their own previous activity	NA	NA	83.78
Zhu et al. (2014)	Dataset 1: 10 Dataset 2: 12	Dataset 1: 10 Dataset 2: 12	rs‐fMRI and DTI	DICCCOLs: 358 ROIs possessing optimized DTI‐derived fiber shape patterns	A two‐stage feature selection procedure was conducted to obtain the most discriminative FC named “connectome signatures.” DICCCOLs that are distributed over the whole cortex offer better functional homogeneity, much finer granularity, more accurate localization functionally, and automatically established cross‐subjects correspondence	NA	NA	Dataset 1:100% Dataset 2:95.8%
Challis et al. (2015)^a^	27	39	rs‐fMRI dataset post‐processed using SBA to include 82 anatomically distinct ROIs based on a priori selection	Gaussian process logistic regression (GP‐LR) model	This dataset also included AD patients. One of the aims was to use the classification to discriminate between MCI and HC	AD versus MCI: 88.0	AD versus MCI: 62.0	AD versus MCI: 77.0
Lee, Ratnarajah, et al. (2015)^a^	61	22	59 brain neural pathways based on a priori knowledge were analyzed 116 nodes were identified and the FC between nodes at paired brain regions was measured by the strength of the linear relationship depicted by *r*	Three linear classifiers: Naïve Bayesian (NB); logistic regression; and SVM One decision trees classifier: RF Diagnostic performances were evaluated on a pathway‐based approach and a region‐based approach	SVM classification model gave the best diagnostic accuracies for discriminating MCI from HC, for both the pathway‐based approach and a region‐based approach.	SVM in Pathway‐based approach:86.0 Region‐based approach:76.0	SVM in Pathway‐based approach:78.0 Region‐based approach:51.0	SVM in Pathway‐based approach:83.0 Region‐based approach:62.0
Suk et al. (2015)	12	25	Group ICA was performed	Linear SVM methods were used ROC curves were plotted	The diagnostic performances of the competing methods were analyzed with HMP and without HMP. The best results were achieved with HMP in regression in the multi‐spectrum analysis	Multi‐spectrum with HMP: 91.67	Multi‐spectrum with HMP: 88.0	Multi‐spectrum with HMP: 89.19
Chen et al. (2017)	54	54	SBA of rs‐fMRI dataset DTI: Static and dynamic functional correlation tensor	SVM and ROC curve Cross‐validation done with LOOCV method	The combined method utilizing static and dynamic FC, FC tensor, gave the best diagnostic performance	Combined multi‐parametric method: 77.78	Combined multi‐parametric method: 79.63	Combined multi‐parametric method: 78.70
de Marco et al. (2017)	50	50	Multiparametric MRI including T1W, T2W, DTI, FLAIR, and rs‐fMRI datasets was analyzed. Neuroanatomic volumetric indices were extracted from the segmentation and parcellation output. FC analyzed based on SBA.	Two types of machine learning algorithms were used: linear and quadratic Fisher discriminant analyses sMRI classifier was heavily reliant upon the right HIPP Other classifiers were cognitive classifiers and rs‐fMRI classifiers	rs‐fMRI+ Cognitive classifier was the most accurate ensemble sMRI classifier was the least accurate	NA	NA	rs‐fMRI wide‐spread connectivity including the medio‐temporal nodes + Cognitive classifier: 94.0
Hojjati et al. (2017)	MCI‐C: 18 MCI‐NC: 62	–	Graph theory was used to calculate different measures of integration and segregation, with 10 local and 13 global graph measures. The integration resulted in a feature vector with 913 elements. [913 = 10 (local measures) × 90 (AAL areas) + 13 (global measures)]	SVM classification was performed Validation method that was used is k‐fold cross‐validation, with *k* = 9 in this study	Three networks were significantly different in the two groups (identified using a threshold at *t* = 9.18 and a *p*‐value < .001). First network comprised four edges and five nodes in bilateral visual cortex (i.e., cuneus) and left language circuit (i.e., opercular part of inferior frontal gyrus and middle temporal gyrus in the left hemisphere. Second network comprised four edges and five nodes, located bilaterally in precuneus as well as in the parahippocampal, fusiform, and superior temporal gyri in the right hemisphere. Third network comprised nine edges and eight nodes, located mostly in the left hemisphere.	MRMR type of SVM classifier: 83.24	MRMR type of SVM classifier: 90.10	MRMR type of SVM classifier: 91.40
Khazaee et al. (2017)^a^	89	45	Graph measure of rs‐fMRI dataset Time series of voxels within each of 264 ROIs were averaged to generate a representative signal for each ROI Binary directed connectivity matrix for each subject was used to calculate 13 graph measures	Multivariate Granger causality is performed by including more than two variables in a MVAR model Types of classifiers used: LDA, KNN, decision trees, SVM, and naïve Bayes classifier were used to discriminate between the features of MCI and HC	Naïve Bayes classifier achieved the best performance to discriminate between the features of MCI and HC, with the top features that had the most discriminating ability rising from nodes of the DMN	SVM: 86.4 Naïve Bayes: 100	SVM: 61.8 Naïve Bayes: 85.5	SVM: 71.95 Naïve Bayes: 93.29
Son et al. (2017)^a^	40	30	10 subcortical regions (thalamus L/R, putamen L/R, hippocampus L/R, caudate L/R, and amygdala L/R) to identify any presence of regional volume atrophy The rs‐fMRI dataset was analyzed using graph theory by using nodes from predefined ROIs and unweighted edges in a square matrix Eigenvector centrality was used as a connectivity measure of the functional networks	Random forest (RF) classifier using identified regional volume and eigenvector centrality values of network functional connectivity as features.	The classifier chose among three possible outcomes and gave improved accuracy. Functional degeneration increased as the disease progressed from HC to MCI to AD, evidenced by 2 regions (putamen L and HIPP R) showed significant differences in volume between HC and MCI. Eigenvector centrality of the HIPP L showed significant differences between HC and MCI.	NA	NA	RF classifier accuracy in distinguishing among HC, MCI, and AD using cortical volume and eigenvector centrality of identified regions: 53.33
Teipel et al. (2017)^a^	84	151	Individual gray matter volumes of the HIPP were extracted. ROIs of brain regions that showed significant group differences in the voxel‐based comparisons of MCI and HC subjects were defined	A block‐wise cross‐validation with repeated random sampling, based on Gaussian‐distributed random numbers generated in R was used to estimate the accuracy of group discrimination for each modality and analysis technique. The dataset was split by a ratio of 3:2 for the training data and the test data, respectively. LR analysis was applied and classification accuracy and AUC were recorded.	MCI versus HC demonstrated peak areas of group effects at the precuneus, MCC, insula cortex, fusiform gyrus, and medial temporal lobes (including amygdala and parahippocampal cortex)	NA	NA	Pooled accuracy: 72.1
Yu et al. (2017)	50	49	Graph theory analysis of rs‐fMRI dataset	Linear SVM classification model was utilized using SGR, WGS, WSR, and WSGR models to select feature indices and perform classification	The proposed brain network construction model (using WSGR) achieved the best classification performance	WSGR: 92.0	WSGR: 76.0	WSGR: 84.85
Zhang et al. (2017)	29	30	Graph theory analysis of rs‐fMRI dataset	LASSO feature selection from various static and dynamic networks; and the weighting factors in the multiple‐kernel learning strategy using SVM classification to discriminate between MCI and HC	Best accuracy of 93.2% is achieved with 1 = 0.3 (for DN_L_), 2 = 0.5 (for DN_H_), and 3 = 1 − (1 + 2) = 0.2 (for DN_A_)	NA	NA	Combined dynamic networks: 93.2
Hojjati et al. (2018)	MCI‐C: 18 MCI‐NC: 62	–	Regional cortical thickness and volumetric measures from the T1‐weighted MRI Graph theory analysis method was used for the rs‐fMRI dataset and the weighted connectivity matrices were converted to binary ones by applying an optimal threshold on the connectivity matrices. A total of 10 local and 13 global graph measures were computed.	Features were extracted from rs‐fMRI based on AAL and Dosenbach atlases separately; and sMRI using the Desikan–Killiany atlas and Destrieux atlas separately. SVM method using a linear kernel was utilized to evaluate the accuracy of the classifiers in discriminating between MCI‐C and MCI‐NC. A subset of features was calculated using the KCV (*k* = 9) cross‐validation approach.	Network‐based statistics were performed on the weighted raw rs‐fMRI connectivity matrices to identify impaired sub‐networks in the MCI‐C and MCI‐NC groups. First network had two edges and three nodes, specifically one node within the precuneus and the other two nodes within the cerebellum. Second network had three edges and four nodes within the vPFC, anterior insula, VFC, and occipital lobe. Third network had two edges and three nodes within the temporoparietal junction, occipital lobe, and lateral cerebellum. Optimal features based on sMRI data using Destrieux atlas and rs‐fMRI data using the Dosenbach atlas gave the best accuracy for discriminating between MCI‐C with MCI‐NC.	Optimal features based on sMRI data using Destrieux atlas and rs‐fMRI data using the Dosenbach atlas: 94.97	Optimal features based on sMRI data using Destrieux atlas and rs‐fMRI data using the Dosenbach atlas: 100.00	Optimal features based on sMRI data using Destrieux atlas and rs‐fMRI data using the Dosenbach atlas: 96.97
Qian et al. (2018)	37	32	Data‐driven method named complementary ensemble empirical mode decomposition (CEEMD) to automatically decompose the BOLD oscillations into several brain rhythms within distinct frequency bands based on GTA	Nonlinear SVM classifier with radial basic function (RBF) kernel was adopted	The most discriminant regions were mainly distributed in paralimbic/limbic and subcortical regions. These regions included the amygdala and ACC, the orbital frontal gyrus deemed to be closely related to olfaction, the HIPP, parahippocampus, putamen, and thalamus that may contribute to cognitive decline in AD. The dysfunction of precuneus and medial SFG, which belong to the regions of DMN, may be associated with the disrupted function of memory retrieval.	NA	NA	CEEMD: 93.33
Hojjati et al. (2019)^a^	MCI‐C: 25 MCI‐NC: 69	49	The adjacency matrix was calculated using the Pearson's correlation between the time series of the fMRI signals of All pairs of 160 ROIs of Dosenbach atlas Converted the weighted adjacency matrices to binary ones by applying An optimal threshold	Discriminant correlation analysis (DCA) and sequential feature collection (SFC) were utilized. The SFC algorithm sorts all features using the multivariate MRMR feature selection algorithm. The MRMR feature selection algorithm selects features that have maximal statistical dependency based on mutual information by considering relevant and redundant features simultaneously The selected features were used to train and cross‐validate an SVM to classify four groups of subjects (AD, MCI‐C, MCI‐NC, and HC) in the train/cross‐validation set.	SFC outperforms DCA for feature selection in three‐ and four‐group classification with an extra accuracy >7%	Discriminating value to discriminate MCI‐NC: Four group classification (AD, MCI‐C, MCI‐NC, HC): 61.8 Three group classification (MCI‐C, MCI‐NC, HC): 71.1	Discriminating value to discriminate MCI‐NC: Four group classification (AD, MCI‐C, MCI‐NC, HC): 72.0 Three group classification (MCI‐C, MCI‐NC, HC): 74.7	Discriminating value to discriminate MCI‐NC: Four group classification (AD, MCI‐C, MCI‐NC, HC): 66.0 Three group classification (MCI‐C, MCI‐NC, HC): 72.0
Lisowska and Rekik (2019)	42	42	For each cortical attribute (e.g., cortical thickness), a single‐view network was constructed for each subject. The network comprised a set of nodes and a collection of edges that connected the nodes (representing the dissimilarity between the two brain regions in morphology). The average value of a cortical attribute was calculated for each anatomical ROI. Six shallow multiplexes were defined, each using two cortical network views. For each cortical attribute, the strength of the morphological network connection linking the *i*th ROI to the *j*th ROI was computed as the absolute difference between the averaged attribute values in both ROIs.	A linear SVM was trained using highly correlated features that were selected from each multiplex. A graph‐guided pairwise group LASSO‐based sparse canonical correlation analysis (GGL‐SCCA) model was utilized to discriminate between early MCI and HC groups	Pericalcarine cortex and insula cortex on the maximum principal curvature view, entorhinal cortex, and insula cortex on the mean sulcal depth view, and entorhinal cortex and pericalcarine cortex on the mean average curvature view for both hemispheres for sMRI data based on FC networks	GGL‐SCCA paired classifier, using shallow convolution identified in: Left cerebral hemisphere: 66.93 Right cerebral hemisphere: 78.59	GGL‐SCCA paired classifier, using shallow convolution identified in: Left cerebral hemisphere: 78.84 Right cerebral hemisphere: 76.19	GGL‐SCCA paired classifier, using shallow convolution identified in: Left cerebral hemisphere: 72.88 Right cerebral hemisphere: 77.38
Jin et al. (2020)^a^	221 MCI subjects (and 252 AD subjects)	215	Four measures of functional brain activity and connectivity derived from each individual's rs‐fMRI data were used: Amplitude Of local brain activity (AM), regional homogeneity (ReHo), functional connectivity strength (FCS) and whole‐brain connectivity	Linear SVM classifier to predict individual diagnostic status, for all patients from the 6 MRI centers, was utilized, combining classifiers from MMSE scores, AM, ReHo, FCS, and whole‐brain connectivity	AD group exhibited significantly lower FC in the insular, compared to MCI and HC subjects	Pooled results based on training dataset: 82.0	Pooled results based on training dataset: 60.0	Pooled results based on training dataset: 70.0
Liu et al. (2020)	Late MCI‐C:105 Early MCI‐NC:105	105	rs‐fMRI data of each subject was parcellated into 78 cortical regions. Two regional network feature sets from rs‐fMRI data for each subject, and denoted as F_CC_ and F_SPL_, respectively. These two‐regional network feature sets are also all 78‐dimensional vectors. sMRI gave two feature sets, that is, F_GMV_ and F_CT_	LASSO regression analysis was used in the feature selection. Multi‐kernel SVM classification was used to find the model that gave the best accuracy to discriminate between late MCI and early MCI compared with HC subjects	The combination of all four sMRI and rs‐fMRI features, that is, (F_GMV_ and F_CT_ F_CC_ and F_SPL_) + MK‐SVM gave the best diagnostic performance to discriminate between the three groups of subjects	Diagnostic performance of combined features based on the classification of: Late MCI/HC: 86.3 Early MCI/HC: 79.4 Late MCI/early MCI: 83.8	Diagnostic performance of combined features based on the classification of: Late MCI/HC: 90.3 Early MCI/HC: 83.9 Late MCI/early MCI: 76.8	Diagnostic performance of combined features based on the classification of: Late MCI/HC: 88.5 Early MCI/HC: 82.7 Late MCI/early MCI: 79.6
Zhang et al. (2020)	82	93	Scale I: Sparsity, classical network metrics for the clustering coefficient (C), characteristic path length (L), global efficiency (GE), and small worldness (SW). Scale II: The regional nodal characteristics regarding the global hubs were assessed qualitatively on the group‐level networks obtained across the sparsities ranging from 5% to 50%. Scale III: The modular structure was evaluated quantitatively via the group‐level networks. The modular organization is one of the most fundamental principles in complex systems. Modularity (denoted as Q), is a measure for the quality of the community structure in a network.	Random Forest approach of machine learning	Scale I: Significantly decreased characteristic path length and increased global efficiency in MCI. Scale II: The nodal betweenness centrality of some global hubs, such as the right Crus II of cerebellar hemisphere and fusiform gyrus changed significantly and were associated with the severity and cognitive impairment in MCI. Scale III: Although anatomically adjacent regions tended to be clustered into the same module regardless of group, discrepancies existed in the composition of modules in both groups, with a prominent separation of the cerebellum and a less localized organization of community structure in MCI compared with NC.	NA	NA	Combining neuro‐psychological assessments and network analysis after feature selection implemented via random forest approach: 91.4

Abbreviations: ACC, anterior cingulate cortex; AUC, area under curve; CAU, caudate; DICCCOLs, dense individualized and common connectivity‐based cortical landmarks; DMN, default mode network; DN_A_, dynamic associated high‐order network; DN_H_, dynamic high‐order network; DN_L_, dynamic low‐order network; F_CC_, feature of clustering coefficient; F_CT_, feature of cortical thickness; F_GMV_, feature of gray matter volume; F_SPL_, feature of shortest path length for a brain network/edge; HC, healthy control; HIPP, hippocampus; HIPP, hippocampus; HMP, head motion profiles; IFG, inferior frontal gyrus; KNN, K‐nearest neighbor; L/R, left and right; LASSO, Least absolute shrinkage and selection operation; LDA, linear discriminant analysis; LING, lingual gyrus; LOOCV, leave‐one‐out cross‐validation; MAR modeling, multivariate autoregressive modeling; MCC, middle cingulate cortex; MRMR, multivariate minimal redundancy maximal relevance; MVAR, multivariate autoregressive; NA, not available; OLS, orthogonal least squares; PCC, posterior cingulate cortex; RSN, resting state network; SFG, superior frontal gyrus; SGR, sparse group representation; sMRI, structural MRI; SVM, support vector machine; VFC, ventral frontal cortex; vPFC, ventral prefrontal cortex; WGS, weighted group sparcity; WSGR, weighted sparsity group representation; WSR, weighted sparse representation.

^a^ Studies that have datasets of both AD and MCI subjects.

Balthazar et al. (2014) evaluated the effect of FC of the DMN, namely impaired FC of the PCC together with cortical atrophy in discriminating 22 patients with mild AD, with 26 age and gender‐matched HC subjects. The authors found a moderate diagnostic power in the FC of the DMN (77.3% sensitivity and 70% specificity) between patients with mild AD and HCs (Balthazar et al., 2014). This indicates that even at the early stage of the AD, DMN moderately differentiates AD patients from HCs. Dai et al. (2012) performed discriminative analysis using FC, ReHo, ALFF, and structural MRI gray matter density (GMD) to differentiate patients with mild AD from HCs. Some DMN nodes (including PCG, mPFC, hippocampus [HIPP], and parahippocampus) were found to be the most prominent distinguishing feature between patients with mild AD and HC (Dai et al., 2012). Additionally, in this study, the diagnostic power was 81.25% sensitivity and 68.18% specificity suggesting a high discriminating power of DMN FC among the patients with AD. de Vos et al. (2018) assessed the DMN impairments in AD patients, in a sample of 70 AD and 173 HCs, using GTA to analyze the FC. They reported a low diagnostic power of DMN between AD and controls with 66 and 67% sensitivity and specificity, respectively (de Vos et al., 2018).

Koch et al. (2012) reported 100% sensitivity and 95% specificity when they utilized ML by combining DMN features from ICA and SBA, to determine the diagnostic power of rs‐fMRI in distinguishing 15 AD patients from 21 HCs. This is an indication that a combination of two methods of analysis yielded a better diagnostic power of DMN abnormalities as a biomarker of AD (Koch et al., 2012). Li et al. (2014) reported that the PCC can serve as a sensitive (73.3%) and specific (93.7%) biomarker for discriminating AD from HC, as shown in ICA analysis of 15 AD patients and 16 HCs (Y. Li et al., 2014). A particular article in our review used Granger causality analysis to study the FC of the DMN among 15 AD and 16 HC subjects, in which they identified impaired connectivity in various DMN hubs among AD subjects (80% sensitivity and 81.25% specificity; Miao et al., 2011). Their results also demonstrated the high diagnostic power of DMN in distinguishing AD from HCs. Furthermore, in their attempt to evaluate the diagnostic power of MRI to detect the alterations in the cortical thickness and DMN FC to classify 41 AD patients and 22 HCs, Park et al. (2017) concluded that both parameters were significant biomarkers of AD, with the latter having 81.3% sensitivity and 74.7% specificity to classify AD from HCs (Park et al., 2017).

In evaluating the difference between patients with early AD and HCs, Yokoi et al. (2018) compared the spatial retention of an amyloid marker, 18F‐THK5351, and the DMN FC. It was noted that the Prec/PCC is a specific hub for retention of 18F‐THK5351 and that the disruption of both the PCC and the DLPFC account for fMRI diagnostic values of 82.6% sensitivity and 79.1% specificity (Yokoi et al., 2018). Zheng et al. (2019) reported the presence of disruption of the FC of the DMN and cerebral blood flow in the brain regions of 40 AD patients compared with 30 HC: namely, in the PCC, DLPFC, inferior parietal lobule (IPL), MTG, MOG, and Prec regions (Zheng et al., 2019). It was observed that the disruption of the FC of the DMN discriminated AD from HCs, with a sensitivity of 65.7% and a specificity of 73.1%. This particular result showed lower discriminating power of fMRI in the classification of AD patients compared to the previously reported results. Furthermore, this drop in FC had a significant positive relationship with the decrease in the patients' MMSE test scores (Zheng et al., 2019).

### 3.4 Types of machine learning methods utilized to classify MCI subjects

The articles that met our inclusion criteria for studying MCI subjects reported the diagnostic power of rs‐fMRI in detecting impaired DMN FC. Koch et al. (2012) indicated that fMRI identified impaired FC of the DMN and correctly classified 17 MCI patients from 21 HCs (Koch et al., 2012). Using the combined methods of ICA and SBA, this yield a sensitivity of 86.5% and a specificity of 85.1%. This result shows that rs‐fMRI has a high diagnostic power in distinguishing MCI from HCs especially when using multiple comparators. Krajcovicova et al. (2017), by utilizing the SBA method, reported a moderate to high diagnostic power of rs‐fMRI to correctly classify MCI patients (sensitivity and specificity of 76.81% and 88.55%, respectively; Krajcovicova et al., 2017). Hojjati et al. (2017) demonstrated that disruptions in several nodes of the DMN can act as a biomarker for classifying MCI patients, giving a sensitivity and specificity of 83.24% and 90.1%, respectively (Hojjati et al., 2017). In several of these studies, rs‐fMRI demonstrated a high diagnostic power in classifying abnormalities of the DMN among MCI patients compared to the HCs (Figure 2).

## 4 DISCUSSION

Although the FC of DMN has been explored as a biomarker for distinguishing patients with AD and MCI from HCs (Brier et al., 2012; Cha et al., 2013; Griffanti et al., 2015), no compiled review about its diagnostic power has been done before this. The impaired FC of DMN may be analyzed using SBA, ICA, and GTA methods of analyses, and all these methods can be used to classify patients with AD and MCI.

To the best of our knowledge, this is the first review to determine the diagnostic power of rs‐fMRI to detect impairments in the FC of the DMN, for discriminating AD and MCI subjects from HCs. The articles included in this review reported variable diagnostic powers of rs‐fMRI in characterizing AD and MCI patients, by using a variety of protocols, that is, measurement of DMN FC alone (Koch et al., 2012; Miao et al., 2011), DMN FC correlated with MRI‐measured cortical thickness (Balthazar et al., 2014; Park et al., 2017), DMN FC measurements along with other resting‐state measures such as DMN FC with PET/CT FC (Yokoi et al., 2018) and DMN FC with regional cerebral blood flow measurements (rCBF; Zheng et al., 2019), respectively.

In differentiating AD patients from HCs, most of the primary articles used SBA analysis, all of which reported that AD patients had weaker FC between the PCC and other brain regions (Balthazar et al., 2014; Dai et al., 2012; Koch et al., 2012; Yokoi et al., 2018; Zheng et al., 2019). This imaging biomarker, that is, the PCC, is able to provide an average sensitivity of 75.2% (ranging between 65.7 and 100%), and an average specificity of 74.9% (ranging between 70 and 95%) for distinguishing patients with AD, hence, indicating a moderate diagnostic power of DMN in differentiating AD patients from HCs. Even though SBA yielded good results, nevertheless, the applicability of solely evaluating the PCC is limited (Koch et al., 2012) because the DMN has numerous hubs that are frequently disrupted in AD (Mohan et al., 2016). Other regions of the DMN that were reported to have weaker FC in AD patients included the ACC, LPC, superior frontal gyrus (SFG), medial temporal cortex (MTC), HIPP, DLPFC, IPL, MTG, MOG, and the Prec. The decreased FC among these regions is consistent with those reported in previous studies (Grieder et al., 2018; Griffanti et al., 2015; Rombouts et al., 2009). The deficient FC within regions of the DMN was correlated with MMSE scores, a global cognition and episodic memory measurement test (Balthazar et al., 2014; Yokoi et al., 2018). Moreover, this pattern of impaired DMN FC is in line with the course of early AD pathology, beginning from the MTG and involving the entorhinal cortex, HIPP, parahippocampus, and fusiform gyri (Du et al., 2004; X. Li, Coyle, Maguire, Watson, & McGinnity, 2011). Advantageously, large‐scale network (LSN) of rs‐fMRI brain networks (DMN and dorsal attention network) can be studied using the ICA method. Articles that met our inclusion criteria, which utilized ICA (Miao et al., 2011; Park et al., 2017), reported that rs‐fMRI had moderate to high diagnostic power to distinguished AD patients from HCs, having an average sensitivity of 78.2% (ranging between 73.3 and 81.3%) and an average specificity of 83.22% (ranging between 74.7 and 93.7%). The average sensitivity and specificity of these results were compared with that of the SBA method. This is likely due to the same data being used for SBA was also used to construct independent component networks of the brain. By combining both the ICA and SBA rs‐fMRI methods and multivariate analysis to evaluate the ACC and PCC, Koch et al. (2012) achieved a high sensitivity and specificity, that is, 100 and 95%, respectively, in discriminating AD from HC subjects (Koch et al., 2012). Apart from the SBA and ICA methods, GTA was an additional method used to analyze fMRI findings in AD, which resulted in a moderate sensitivity of 67% and specificity of 66%, respectively (de Vos et al., 2018).

In comparison, Koch et al. (2012) failed to produce any statistical difference between the FC of the DMN regions, that is, in the ACC, PCC, LPC, SFG, MTC, and HIPP, of the AD and HCs. They also reported a lower diagnostic power of rs‐fMRI, that is, sensitivity of 64.7% and specificity of 95.2%, respectively (Koch et al., 2012). Lee et al. (2015) using SVM determined the diagnostic power of rs‐fMRI to detect DMN FC abnormalities in classifyingaMCI from HCs. Their method achieved a high diagnostic power, with a sensitivity of 86% and specificity of 78%, respectively, with better results achieved when using the pathway‐based approach compared with the region‐based approach for classifying MCI from HCs (Lee et al., 2015).

In essence, rs‐fMRI can detect impairment of the DMN FC and can serve to identify important anatomical biomarkers for discriminating AD and MCI patients from HCs. When combined with other parameters such as cortical thickness, rCBF, or analyzed using combination of multivariate analysis, rs‐fMRI has good diagnostic power for detecting AD and MCI.

### 4.1 Limitations and recommendations for future works

The relatively small sample size in most of the articles leads to a reduced power of the studies. Restrictions of the studies to only include subjects with early AD had to be made due to the constraints of performing the investigation on non‐cooperative patients with advanced AD. Furthermore, it is important to note that although MCI may occur as a prodromal condition to AD, it can also occur in vascular dementia or even in cognitively healthy elderly persons without progressing to AD. Moreover, the conversion rate of MCI to AD is usually meager. Therefore, longitudinal studies, as opposed to identifying neural FC changes using a single time‐point rs‐fMRI study, can best assess whether an MCI patient will develop full‐blown AD. Additionally, there is a need for further improvement and standardization of rs‐fMRI patient selection criteria, acquisition, image‐processing, and data analysis. The establishment of local population‐based database of fMRI studies involving AD subjects can also help in improving the suitability of comparison. Multicenter rs‐fMRI using SBA FC has limited accuracy in the discrimination of AD and MCI cases from HC and requires careful data quality checks beyond the evaluation of global quality metrics, including visual inspection of all the data (Teipel et al., 2017). Furthermore, the combination and integration of multimodal imaging and clinical markers, introduce innumerable classifiers for the improved diagnostic accuracy of detecting and predicting AD. Although it appears very enticing to incorporate numerous multimodal features, nevertheless, this poses a challenge for ensuring homogeneity of datasets and hinders consistency of results. Other rs‐fMRI features of engineering models that go beyond the classical Pearson correlation FC and ICA, that is, regional homogeneity (ReHo), fractional amplitude of low‐frequency fluctuation ((f)ALFF), and dynamic FC need to be explored further to optimize the wealth of information available on rs‐fMRI datasets. Additionally, novel computational models using convolutional neural networks that use 3D‐deep learning frameworks are the way forward. There is potential for developing the utility of this technique by incorporating biomarker‐based serial labeled data and domain transfer learning methods.

## 5 CONCLUSION

The assessment of the DMN FC based on rs‐fMRI analytic methods, has an excellent potential as a diagnostic tool for AD, particularly when using multivariate analysis to combine SBA and ICA methods of analyses. Nevertheless, the rs‐fMRI protocols and analytical methods need to be more standardized to achieve uniformity in reporting improved diagnostic power.

## CONFLICT OF INTEREST

The authors declare and report no conflict of interest.

## AUTHOR CONTRIBUTIONS

Subapriya Suppiah conceptualized the study design. Buhari Ibrahim and Nisha Syed Nasser carried out the literature search, data extraction, and quality assessment. Buhari Ibrahim wrote the manuscript first draft. Subapriya Suppiah, Normala Ibrahim, Mazlyfarina Mohamad, Hasyma Abu Hassan, and M Iqbal Saripan edited the manuscript, verified the data, and provided critical feedback to help shape the research.

## Supporting information


**Table S1** List of the criteria used for assessing the methodological quality

## Data Availability

Data sharing is not applicable to this article as no new data were created or analyzed in this study.
