# Erratum

**DOI:** 10.1002/hbm.25704

**Published:** 2021-11-01

In the article titled “Diagnostic power of resting‐state fMRI for detection of network connectivity in Alzheimer’s disease and mild cognitive impairment: A systematic review” by Buhari Ibrahim,Subapriya Suppiah, Normala Ibrahim, Mazlyfarina Mohamad, Hasyma Abu Hassan, Nisha Syed Nasser, M Iqbal Saripan (Human Brain Mapping, 2021, Vol 42 pp. 2941–2,968, https://doi.org/10.1002/hbm.25369), there was an error in the acknowledgement /funding information section. The acknowledgement omitted the following: “The research grant was awarded to Associate Professor Dr Subapriya Suppiah by the Malaysian Ministry of Education, that is, the Fundamental Research Grant Scheme (FRGS) with a grant number 5540244 and reference code number FRGS/1/2019/SKK03/ UPM/02/4, helped support this research. (Geran Kementerian Pendidikan Malaysia, nombor kod rujukan FRGS/1/2019/SKK03/UPM/02/4)”.

The acknowledgement section should have instead read as the following: “This research was funded by the Ministry of Higher Education, Malaysia under the Fundamental Research Grant Scheme (FRGS/1/2019/SKK03/UPM/02/4) and project code: 04‐01‐19‐2119FR and project number 5540244 that was awarded to Associate Professor Dr. Subapriya Suppiah (Geran Kementerian Pengajian Tinggi, Malaysia, nombor kod rujukan FRGS/1/2019/SKK03/UPM/02/4 dengan nobor projek 5540244).”

