# Supplementary material for: Diagnostic power of resting‐state fMRI for detection of network connectivity in Alzheimer's disease and mild cognitive impairment: A systematic review
Source: Hum Brain Mapp. 2021 May 4;42(9):2941–68. doi: 10.1002/hbm.25369 (PMC8127155; doi:10.1002/hbm.25369)
Supplement: Supplementary file 1 — Table S1 List of the criteria used for assessing the methodological quality [file HBM-42-2941-s001.docx]

**Supplementary Table**

**Supplementary Table: list of the criteria used for assessing the methodological quality**

| **S/N** | **Quality assessment** | **Positive response** |
| --- | --- | --- |
| 1 | Was the spectrum of patients representative of the patients who will receive the test in practice? | Only AD and MCI patients were included and identified based on established diagnostic criteria either by DSM-IV or revised NINCDS–ADRDA |
| 2 | Were selection criteria clearly described? | It was clear how patients were selected for fMRI imaging |
| 3 | Is the reference standard likely to enable correct classification of the target condition? | Only clinical probable diagnosis of AD and MCI based on established diagnostic criteria either by DSM-IV or revised NINCDS–ADRDA is considered the reference standard |
| 4 | Did the whole sample or a random selection of the sample receive verification with a reference standard? | All patients received verification with DSM-IV or revised NINCDS–ADRDA is considered the reference standard |
| 5 | Did patients receive the same reference standard regardless of the index test result? | All patient received the same reference standard |
| 6 | Was the reference standard independent of the index test (the index test did not form part of the reference standard)? | fMRI did not form part of the reference standard |
| 7 | Was the execution of the index test described in sufficient detail to permit replication of the test? | The fMRI protocol including scanning time, type of scanner and acquisition mode were described in detail. |
| 8 | Was the execution of the reference standard described in sufficient detail to permit its replication? | Adequate information or citation on the reference standard was given |
| 9 | Were the index test results interpreted without knowledge of the results of the reference standard? | Interpretation of the fMRI results was done without the knowledge of the reference standard |
| 10 | Were the reference standard results interpreted without knowledge of the results of the index test? | Interpretation of the reference standard was done without the knowledge of the result of the index test |
| 11 | Were the same clinical data available when test results were interpreted as would be available when the test is used in practice? | All clinical and demographic characteristic of the patients was available when the test result was interpreted |
| 12 | Were uninterpretable and ⁄ or intermediate test results reported? | All the fMRI results (uninterpretable and /or intermediate) were reported |
| 13 | Were withdrawals from the study explained? | The reason for subject withdrawal was reported |
|  |  |  |
